# A Fully Automated Conversational Artificial Intelligence for Weight Loss: Longitudinal Observational Study Among Overweight and Obese Adults

**DOI:** 10.2196/diabetes.8590

**Published:** 2017-11-01

**Authors:** Natalie Stein, Kevin Brooks

**Affiliations:** 1 Division of Public Health College of Human Medicine Michigan State University Flint Campus, MI United States; 2 Division of Public Health College of Human Medicine Michigan State University East Lansing, MI United States

**Keywords:** obesity, artificial intelligence, self efficacy, weight loss, prediabetes, smartphone, diabetes, compassion, mobile health, text messaging

## Abstract

**Background:**

Type 2 diabetes is the most expensive chronic disease in the United States. Two-thirds of US adults have prediabetes or are overweight and at risk for type 2 diabetes. Intensive in-person behavioral counseling can help patients lose weight and make healthy behavior changes to improve their health outcomes. However, with the shortage of health care providers and associated costs, such programs do not adequately service all patients who could benefit. The health care system needs effective and cost-effective interventions that can lead to positive health outcomes as scale. This study investigated the ability of conversational artificial intelligence (AI), in the form of a standalone, fully automated text-based mobile coaching service, to promote weight loss and other health behaviors related to diabetes prevention. This study also measured user acceptability of AI coaches as alternatives to live health care professionals.

**Objective:**

The objective of this study was to evaluate weight loss, changes in meal quality, and app acceptability among users of the Lark Weight Loss Health Coach AI (HCAI), with the overarching goal of increasing access to compassionate health care via mobile health. Lessons learned in this study can be applied when planning future clinical trials to evaluate HCAI and when designing AI to promote weight loss, healthy behavior change, and prevention and self-management of chronic diseases.

**Methods:**

This was a longitudinal observational study among overweight and obese (body mass index ≥25) participants who used HCAI, which encourages weight loss and healthy diet choices through elements of cognitive behavioral therapy. Weight loss, meal quality, physical activity, and sleep data were collected through user input and, for sleep and physical activity, partly through automatic detection by the user’s mobile phone. User engagement was assessed by duration and amount of app use. A 4-question in-app user trust survey assessed app usability and acceptability.

**Results:**

Data were analyzed for participants (N=70) who met engagement standards set forth by the Centers for Disease Control and Prevention criteria for Diabetes Prevention Program, a clinically proven weight loss program focused on preventing diabetes. Weight loss (standard error of the mean) was 2.38% (0.69%) of baseline weight. The average duration of app use was 15 (SD 1.0) weeks, and users averaged 103 sessions each. Predictors of weight loss included duration of AI use, number of counseling sessions, and number of meals logged. Percentage of healthy meals increased by 31%. The in-app user trust survey had a 100% response rate and positive results, with a satisfaction score of 87 out of 100 and net promoter score of 47.

**Conclusions:**

This study showed that use of an AI health coach is associated with weight loss comparable to in-person lifestyle interventions. It can also encourage behavior changes and have high user acceptability. Research into AI and its application in telemedicine should be pursued, with clinical trials investigating effects on weight, health behaviors, and user engagement and acceptability.

## Introduction

### The Burden of Type 2 Diabetes

An estimated 30.3 million Americans, or 9.4% of the US population, have type 2 diabetes (T2D). Another 84.1 million, or 33.9% of the adult US population, has prediabetes and is at risk for developing T2D [1]. The estimated cost of diabetes in 2012 was US $245 billion [2]. Another estimated cost is an extra annual per-patient cost of US $4217 [3]. It is the country’s most expensive chronic disease [4], the seventh-leading cause of death in the United States, and a risk factor for complications and cardiovascular disease [2].

The T2D burden is largely attributable to modifiable risk factors [5]. Each 1 kg decrease in excess body weight lowers T2D risk by 16% among individuals with prediabetes [6]. Modest weight loss among overweight individuals also improves glycemic control [7-10]. Other modifiable risk factors for T2D include diet quality, sleep [11], and physical activity [12]. Diets rich in fruit, vegetables [13-15], low-fat dairy products, polyunsaturated fatty acids, nuts, dietary fiber, and whole grains [16], and diets lower in red and processed meat, refined grains, and sugar-sweetened beverages can lower T2D risk [17,18]. Still, two-thirds of American adults are overweight or obese [19]. Consumption of nutrient-dense foods, such as vegetables, fruits, whole grains, seafood, and low-fat dairy products is low while consumption of solid fats and added sugars is high [20]. Nearly 4 out of 5 adults fail to meet physical activity recommendations for aerobic and strength training exercise [21].

### Lack of Health Care System Resources

Lifestyle modification programs can lead to weight loss and reduction of diabetes risk [22], but they are difficult to maintain on one’s own [23], and health care resources are limited [24]. The Association of American Medical Colleges projects shortfalls of both primary care physicians and endocrinologists by 2030 [25]. Both providers and patients report lack of time [26]. Patients also perceive a lack of provider compassion, including components such as sensitivity, caring, and understanding [27], despite both the Health and Medicine Division, formerly the Institute of Medicine, and the American Diabetes Association recognizing the significance of patient-centered care [28,29].

Economic resources in the health care system are inadequate for preventive measures such as weight loss and other behavioral changes. Diabetes with complications is among the most expensive condition billed to Medicare [27], and most of the T2D expenditures in the United States are for intensive treatments such as hospital inpatient care (43%), prescription medications to treat complications (18%), and nursing/residential facility stays (8%) [2].

### The Role of Artificial Intelligence in Compassionate Diabetes Care

Significant progress has been made in leveraging technology to increase efficiency and improve health outcomes, including in chronic disease self-management [30]. For example, mobile apps have been used for patient monitoring, health service support, treatment, diagnosis, health promotion, and disease prevention [31], and to support weight and diabetes management and glycemic control [32-35]. However, while technology has improved health care efficiency, such as in the use of electronic health records [36], it has not been widely used to increase compassionate patient-centered care in direct patient interactions despite evidence that empathy and patient-centered care result in better health outcomes [37]. Technology such as artificial intelligence (AI) can help fulfill this need in T2D prevention by promoting healthy lifestyle changes. By utilizing AI that is compassionate and effective, these programs can reduce the need for in-person appointments and direct patient-provider interaction, providing much-needed scalability to relieve pressure on limited health care resources.

As AI and mobile health technology provide a platform to make health behavior coaching programs more accessible to patients, they can also enable the scaling up of empathy and compassion. It can be designed to be compassionate based on characteristics defined in the literature, such as being one-on-one, individualized, and responsive to patients, and having “empathy plus sympathy” [38]. Scalable technologies such as conversational AI can have a notable impact on T2D prevention and management, in which sustained patient self-efficacy and behavior change greatly affect health outcomes.

### The Lark Health Coach Artificial Intelligence Mobile Phone App

The Lark Health Coach AI (HCAI) mobile phone app was designed with goals of achieving healthy behavior change among at-risk users and introducing compassionate care in health care systems to allow patients access to infinitely scalable healthy behavior change coaching and support.

Lark’s AI health coaches mimic health professionals’ empathetic health counseling through casual conversations using empathetic text-based communication and other interactive elements. Lark has a variety of products focusing on chronic conditions including obesity and diabetes. In this study, we looked at Lark’s Weight Loss HCAI, which is a product focused on promoting weight loss and other diabetes-preventing and diabetes-managing behaviors such as achieving and/or maintaining healthy sleep duration [11], choosing foods and beverages categorized as “healthy,” and setting and achieving daily and weekly activity goals. Portions of the HCAI use content and methods based on the Diabetes Prevention Program (DPP) curriculum, which has been shown to lead to weight loss [39].

The HCAI aims to increase compassion in health care according to the definition of compassion: “the feeling that arises in witnessing another’s suffering and that motivates a subsequent desire to help” [40]. The AI witnesses user suffering or positive feelings through user-provided feedback about their struggles, such as not losing weight or feeling ill, or accomplishments, such as eating a healthy meal. It shows a “subsequent desire to help” through conversations around the event or feeling. The conversations deliver messages that provide both strategic help (eg, “A pastry here and there won’t hurt you/But next time experiment with which healthier alternatives you also find yummy and satisfying”), as well as emotional support (eg, “know that when it comes to weight loss, ups and downs are typical”).

To promote sustainable behavior change and increased self-efficacy, the AI incorporates interactive elements of cognitive behavioral therapy (CBT) such as reflection, legitimization, respect, support, and partnership [24]. Conversations with patients are triggered by and responsive to real-time data gathered automatically from sensors on phones or integrated devices such as wearables, or by patient-provided information, such as dietary consumption. Features intended to improve the user experience include the continuous and unlimited availability of the app, as well as responsiveness to user input. Users can initiate conversations with their health coach any time and receive immediate feedback. The health coach responds to users’ specific input, such as food and beverage consumption, weight, or sleep duration, with relevant content such as praise, educational material, or reflection.

### Study Overview and Aims

Because of the shortcomings in traditional health care delivery channels to help patients achieve healthy lifestyle changes for lowering T2D risk, and the potential for mobile technologies to provide effective and compassionate interventions, there is a role for conversational AI to provide highly scalable health coaching to effect positive change in behaviors known to lower T2D risk. This study’s objectives were to (1) investigate conversational AI use and relationships with weight loss and meal healthiness, and (2) investigate user engagement and acceptability of the HCAI. We hypothesized that AI users would lose weight and improve meal healthiness.

## Methods

### Study Design

#### Recruitment

This was a retrospective study among 239 overweight and obese (body mass index [BMI] of at least 25 kg/m^2^) adults at one of six primary care offices in Nevada and southern California who were within a provider network that had partnered with Lark for this trial. Patients’ primary care physicians offered the HCAI free of charge to patients meeting the BMI requirement. Additional selection criteria were use of Android or iOS mobile phones and not being previous or current Lark app users. Patients who agreed to use the app had a link to install the app sent to their mobile device during the office visit. No further physician support was provided to patients. Initial use of the app took place from July 2016 to January 2017.

#### Ethics

Michigan State University’s Institutional Review Board (IRB) determined that this study was not classified as human subject research and therefore did not require IRB approval.

### Lark Health Coach Artificial Intelligence Mobile Phone App

#### Health Coach Development and User Setup and Experience

Lark (Lark Technologies) HCAI has been available for Android and Apple mobile phones since 2015. The HCAI provides weight loss coaching through modules with lessons on topics such as self-monitoring, goal-setting, and action planning, plus unlimited text-based quick counseling sessions to help users achieve behavior change goals. Users can complete the modules within 16 weeks, or they can take longer if they repeat lessons or avoid logging into the app for a week or more. The HCAI learns about users and provides personalized content. Additional human-like coaching aspects include guiding dialogues with users and leading users through goal-setting modules for weight loss and food choices.

**Figure 1:**
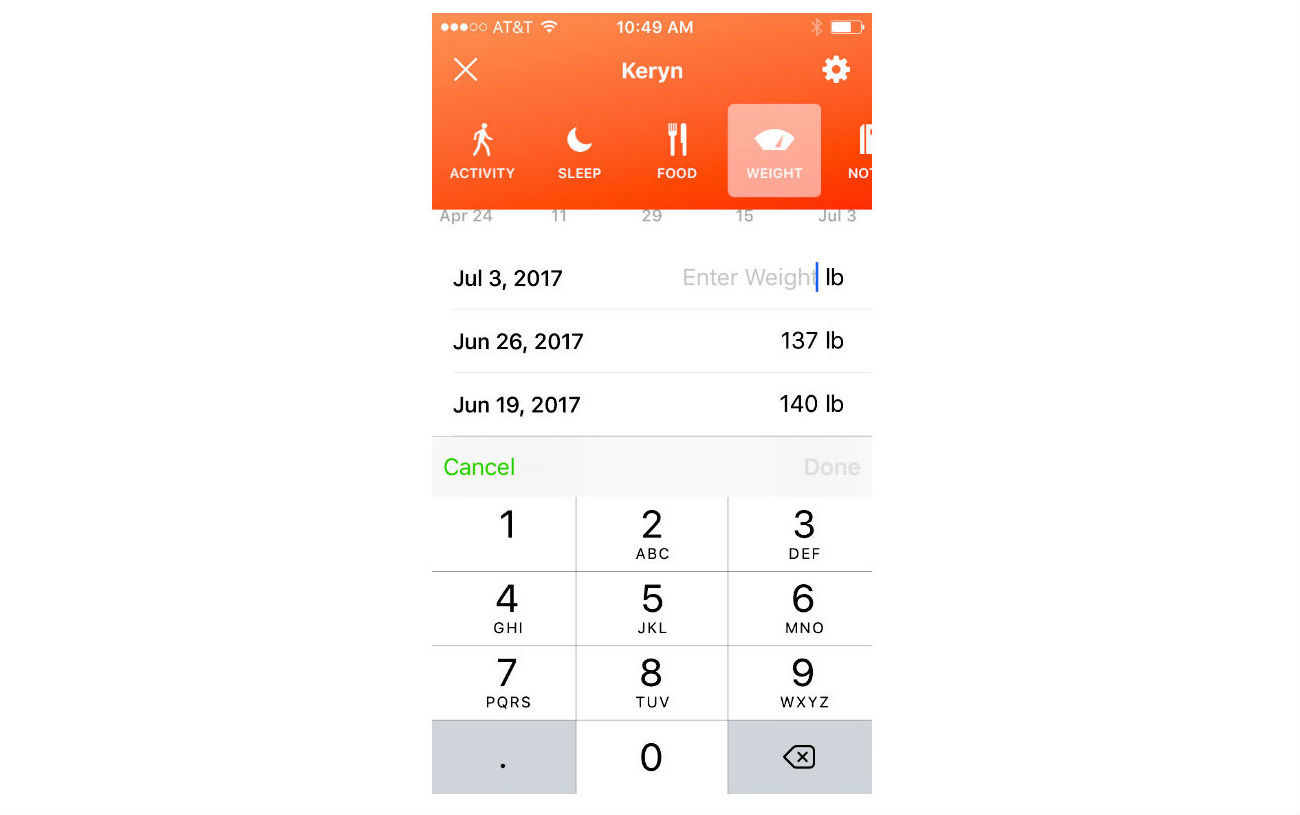
User weight progress dashboard, where users can enter weight (left two panels) and see a chart of weight change since starting the program (right panel).

**Figure 2:**
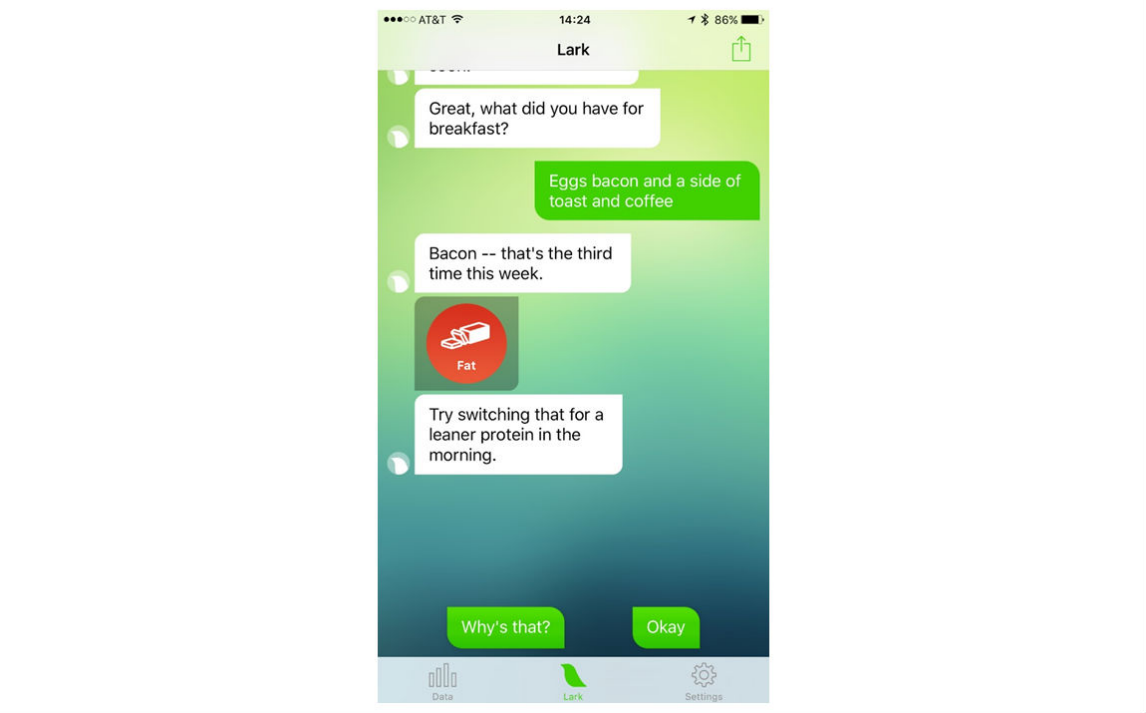
Sample portion of a conversation with the AI promoting healthy behavior change through compassion and cognitive behavioral therapy strategies including in-the-moment responsiveness, responsiveness to user input, and reflection.

**Figure 3:**
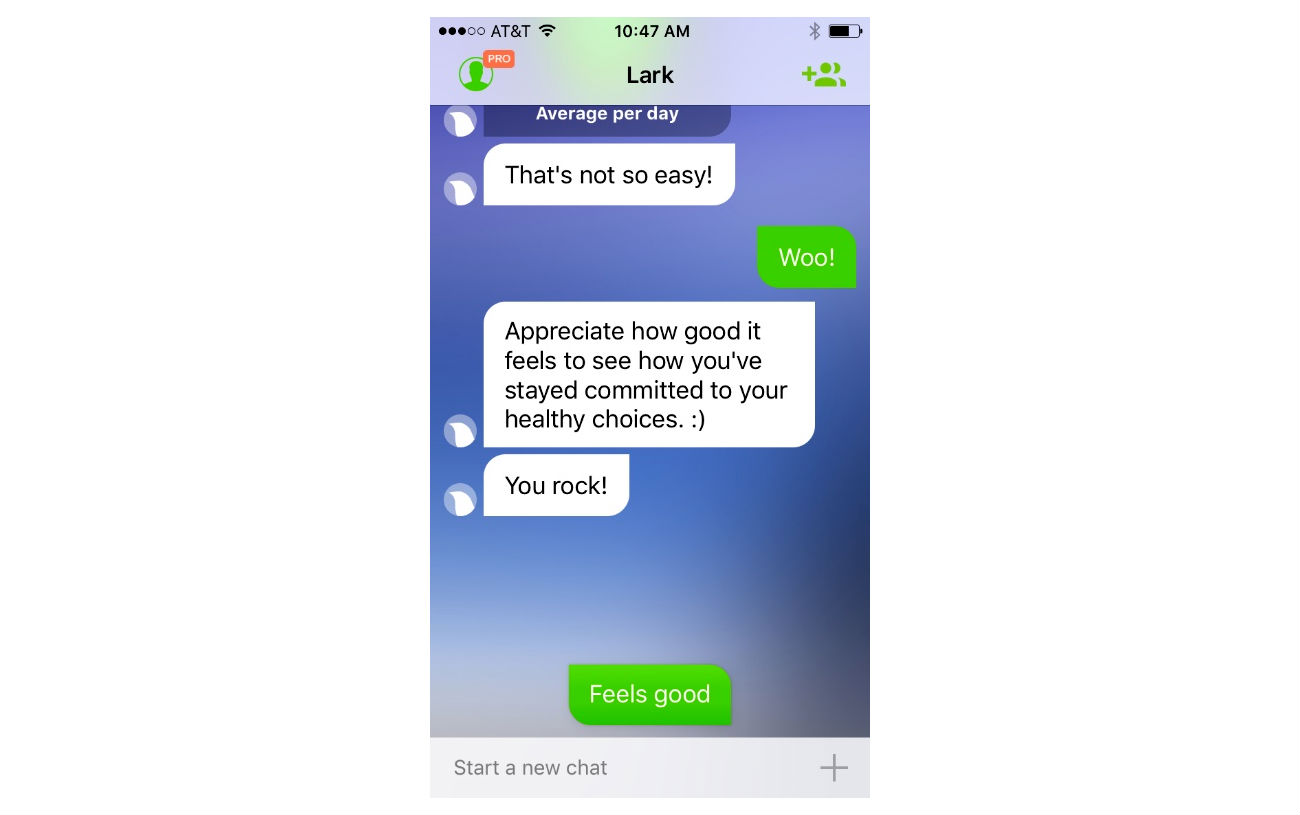
Sample conversations about goal weight and user weight loss.

**Figure 4:**
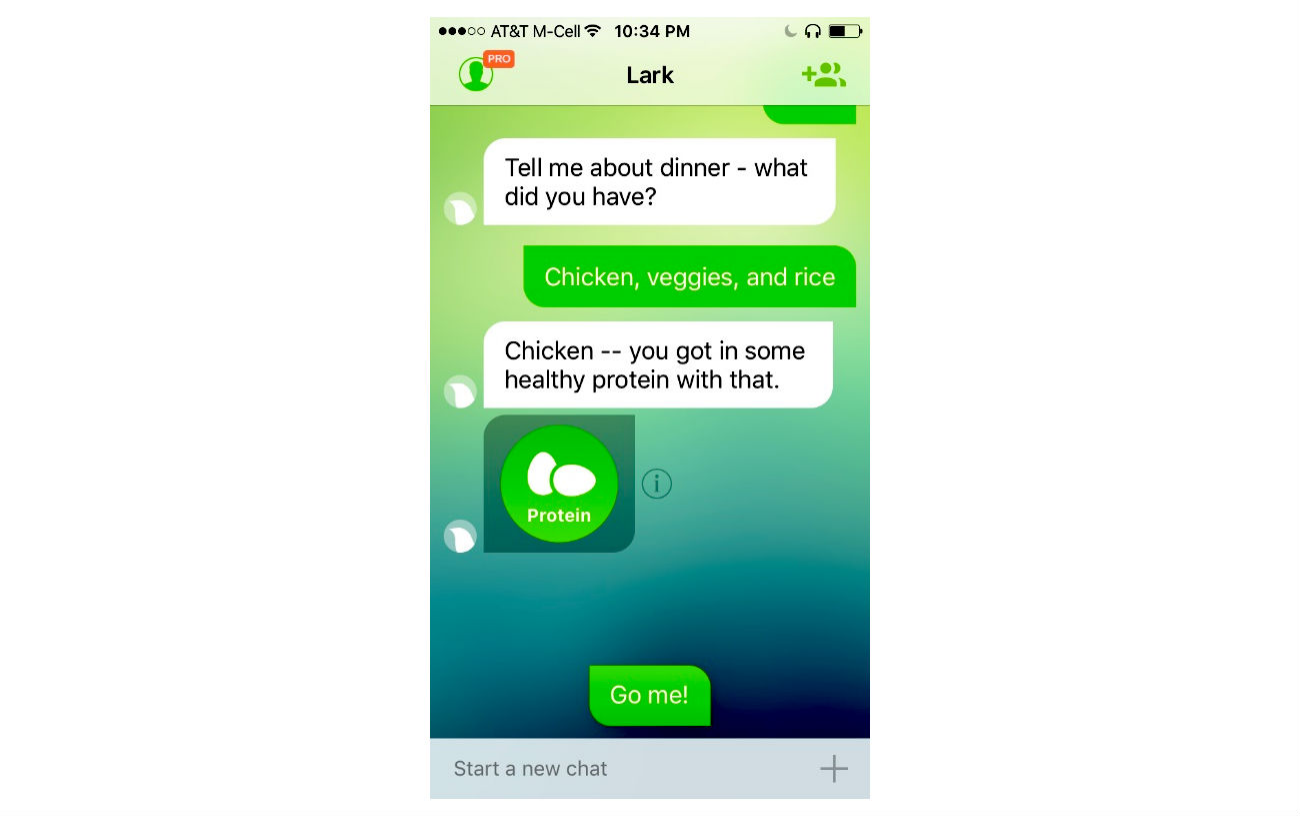
Sample conversations following user-logged meals.

**Figure 5:**
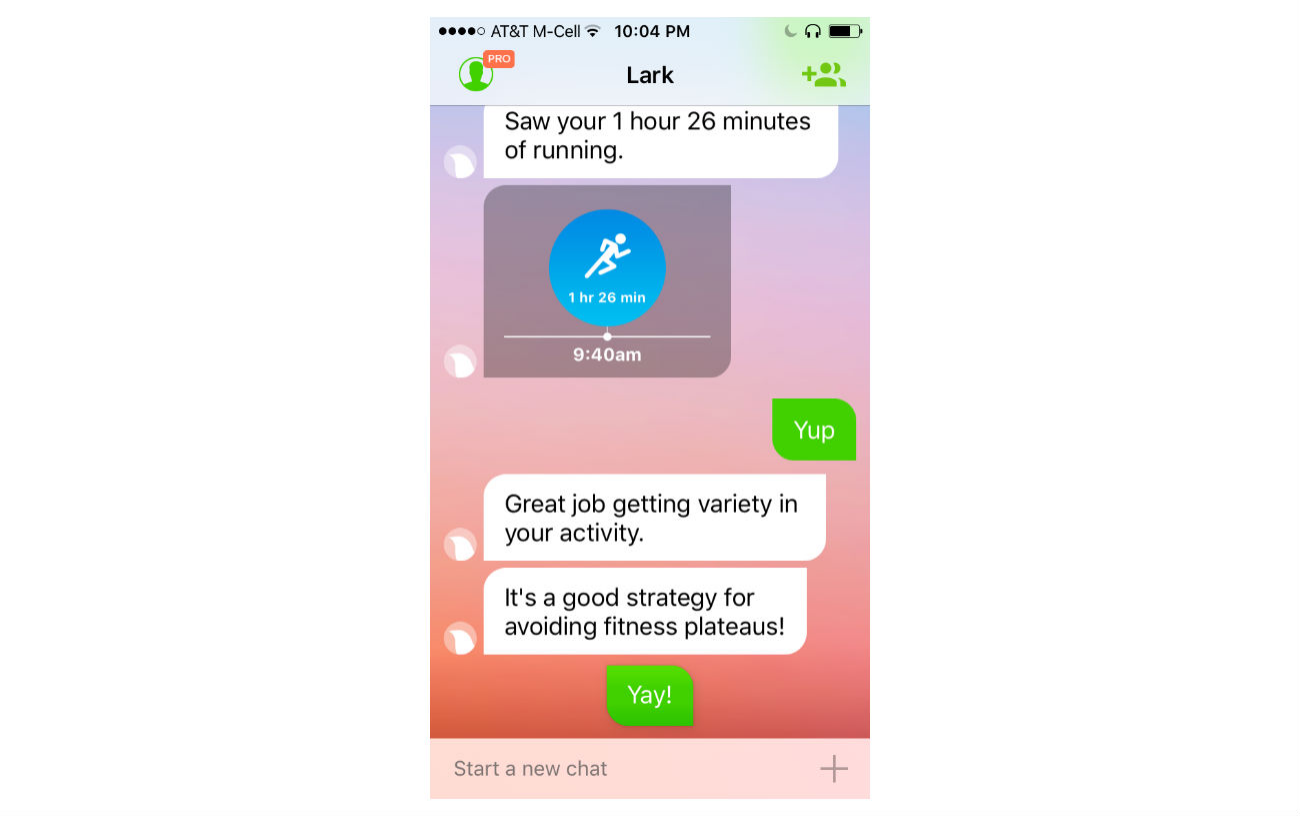
Conversation following user-logged bout of physical activity (1 hour, 26-minute run) praising the user for the run, informing the user (left panel) that the run is a good strategy for increasing overall activity, and (center and right panels) comparing the user’s total current activity for the day (green line) to the user’s daily average on weekend days since starting the program (white dashed line).

When setting up the app, users are asked to enter age, gender, weight, and height, and are guided through content to set a goal weight. Users can choose their goal weight but are discouraged from selecting a goal weight that would put them at an underweight BMI (≤18.5 kg/m^2^). The HCAI prompts users to enter their weight weekly and to enter meals and snacks. They can also enter their weight measurements and diet consumption anytime (Figure 1). Users receive feedback immediately after data entry and in daily and weekly update conversations. Users can log in to the app and initiate conversations at any time. Conversations are designed to look like standard text message conversations (Figures 2-5).

### Outcome Evaluation

#### Weight Loss

Each user’s weight loss was calculated as the difference between the final recorded weight and the baseline weight. The primary outcome in this study was percent weight change.

#### Meal Quality

The HCAI classified individual foods and beverages as “healthy” if they promote weight control based on literature, or they were nutrient-dense or have predominantly nutrient-dense components (eg, vegetables, whole grains, fruit, nuts, lean proteins, and mixed foods such as vegetarian burgers and Greek salad); “unhealthy” if associated with weight gain based on literature and/or contain many empty calories [20] (eg, fried foods, sweets, sugar-sweetened beverages, and fatty red and processed meats); or “neutral” if not classified as “healthy” or “unhealthy” (eg, corn, which is high in nutrients such as dietary fiber and potassium, but higher in starch and calories than many other vegetables). Meals were recorded as “healthy” if they contained at least one healthy food and no unhealthy foods, and “unhealthy” if they contained at least one unhealthy and no healthy foods.

Percent healthy and unhealthy meals at baseline were calculated by dividing the total number of healthy and unhealthy, respectively, meals logged by the total number of meals logged (including healthy, unhealthy, and neither) during the first week of logging. Final percent healthy and unhealthy meals were calculated based on the final week that users logged meals.

#### User Engagement

Duration of AI use was measured by the time, in weeks, between a user’s first and final use of the app. The number of conversations each user had with the app was also recorded.

#### Artificial Intelligence Acceptability and User Satisfaction

User satisfaction was assessed by an in-app user trust survey with four questions measuring (1) overall satisfaction, (2) net promoter score (NPS), (3) disappointment if HCAI were not oﬀered, and (4) self-reported health improvement (Table 1). The satisfaction score (SS; Question 1) was the percentage that rated satisfaction as 6-10. Question 2 was used to calculate NPS by subtracting the percentage of detractors (score 0-6) from the percentage of promoters (score 9-10), as described by Krol et al [41]. The disappointment score (DS; Question 3) was the percentage that rated disappointment if the HCAI were not offered as 6-10. Health outcome score (HOS) was percentage of users responding that their health was “Much better than before” or “Somewhat better than before.” The SS, DS, and HOS were developed directly with the provider network.

**Table 1:** User trust survey to determine patient SS, NPS, DS, and HOS.

Measurement	Question text
SS	How would you rate your overall satisfaction with the Lark Weight Loss Program (where 10 is Very Satisﬁed and 0 is Very Dissatisﬁed)?
NPS	How likely are you to recommend the Lark Weight Loss Program to others (where 10 is Extremely Likely and 0 is Extremely Unlikely)?
DS	If the need were to arise again in the future, how disappointed would you be if the Lark Weight Loss Program was not available to you (where 10 is Extremely Disappointed and 0 is Not at all disappointed)?
HOS	As a result of the help you received from the Lark Weight Loss Program, would you say your health is (Much better than before, Somewhat better than before, Neither better nor worse, Somewhat worse, Much worse than before)?

### Statistical Analysis

#### Dataset

**Figure 6:**
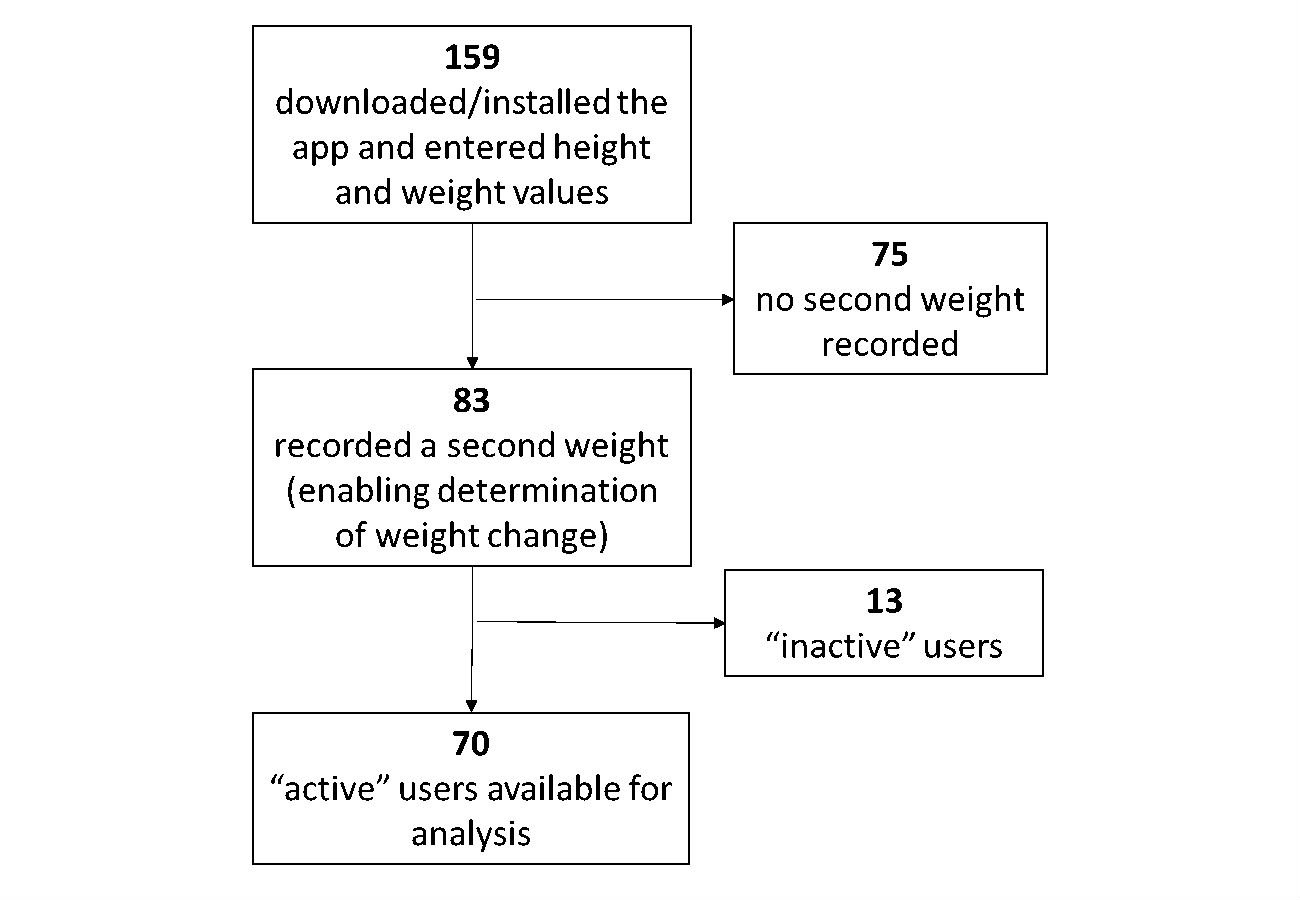
Participant selection flow. “Active” users recorded conversations with the HCAI in at least 4 separate weeks.

Data points were user-entered values for age, gender, height, weight, dietary intake, with self-reported anthropometric data [42,43] and Web-reported diet intake previously validated [44,45]. As shown in Figure 6, participants installed the app and provided their height and baseline weight. An additional 81 additional participants had downloaded the app, making an initial total of 239, but they failed to provide initial height and/or weight data. Data from another 76 participants were eliminated from analysis for failing to record a second weight, making it impossible to determine weight change from baseline. Consistent with guidelines used to evaluate DPP outcomes [46], 13 of the remaining 83 users were recorded as “inactive” due to failure to record conversations with the HCAI in at least 4 separate weeks, leaving data from 70 participants available for analysis.

#### Missing Data

The age variable had 27 missing values, so ages were imputed according to accepted methods [47]. Only instances where the variable age had missing values were imputations applied and outliers removed. There was no significant difference between the new and existing variables for age.

#### Data Analysis

We examined associations between percent weight loss and a set of selected independent variables using univariate and multivariate analyses. Variables determined to be statistically significant at an alpha of .2 in the univariate analysis were selected in multivariate analyses to control for the effects of other variables. Variables were also assessed for collinearity using variance inflation factor. Generalized regression was used to quantify the independent association between selected covariates and percent change in weight. We applied a weighting factor consisting of the number of entries made per user to normalize the associations. All statistical analyses were conducted using JMP Pro, Version 13.1.0. SAS Institute Inc.

## Results

### User Statistics

Participant baseline characteristics are presented in Table 2. Users were 74.5% (35/47) female with average age 47 years. Baseline weight was 98.0 kg (SD 3.16) and BMI was 37.0 kg/m^2^ (SD 1.40). Standard error of the mean (SEM) is included.

### Weight Loss, Meal Quality, User Engagement, and Artificial Intelligence Acceptability and User Satisfaction

Users averaged 103 sessions each over the course of 15.0 weeks, where a session constituted a discrete text-based conversational interaction between the user and the HCAI. Users averaged 2.4 kg or 2.4% weight loss (Table 3), and 75.7% (53/70) of users lost weight in the program. The percentage of healthy meals increased by 31% (from 51% [414/808] of total meals logged at baseline to 67% [22/33] at 21 weeks), and the percentage of unhealthy meals decreased by 54% (from 14% [117/808] to 6% [2/33]). User height, baseline weight, number of conversations with the HCAI, total number of meals logged, and numbers of healthy and unhealthy meals were associated with weight loss (*P*<.25) (Table 4). The number of conversations a participant had with the HCAI was also associated with weight loss when combined with duration of use. The total number of meals logged was a significant predictor of weight loss, while the number of unhealthy meals logged was a significant predictor of weight gain. Gender was statistically significant but not included in the multivariate model due to small sample size. Number of healthy meals logged was removed to avoid collinearity with number of meals logged. The variance inflation factor between these variables was greater than 10 [48].

**Table 2:** Baseline characteristics of app users (N=70)^a^.

Variables	Mean (SEM)	95% CI	Range
Age, years	46.9 (1.89)	43.1 to 50.7	18 to 76
Height, cm	163 (1.41)	161 to 167	135 to 188
Baseline weight, kg	98.0 (3.16)	91.7 to 104	55 to 219
Baseline BMI, kg/m^2^	37.0 (1.40)	34.1 to 39.9	24 to 95

^a^Eight lower outliers were replaced with 1.5 sigma of smallest height value without outliers.

**Table 3:** Weight change and HCAI use (N=70).

Variable	Mean (SEM)	95% CI	Range
Final weight, kg	95.7 (3.20)	89.3 to 102	54 to 220
Final BMI, kg/m^2^	36.0 (1.44)	33.2 to 38.9	24 to 95
Weight change, kg	-2.40 (0.82)	-4.03 to -0.77	-54 to 5
Weight change, %	-2.38 (2.4/98) (0.69)	-3.75 to -1.00	4 to 44
Duration of AI use in weeks	15.0 (1.0)	13.1 to 17.0	4 to 33
Number of conversations with AI	103 (13.8)	75.0 to 130	5 to 824
Number of weight entries	6.1 (0.6)	5.0 to 7.3	2 to 32
Number of meals logged	68 (8.5)	49.8 to 84.7	0 to 351
Healthy meals logged, %^a^	59% (40.2/68) (5.71)	28.9 to 51.7	0 to 247
Unhealthy meals logged, %^b^	11% (7.54/68) (1.16)	5.24 to 9.85	0 to 53

^a^Eight lower outliers were replaced with 1.5 sigma of smallest height value without outliers.

^b^The percent of healthy plus unhealthy meals does not total 100% because some meals were categorized as neither healthy nor unhealthy.

**Table 4:** Factors correlated with weight loss.

Variable	Univariate linear regression^a^	*P*	Multivariate generalized regression^a^	*P*
	*β* (95% CI)		*β* (95% CI)	
Gender^b^	1.52 (-0.30 to 3.34)	.10		
Age, years	0.02 (-0.021 to 0.056)	.365	0.082 (0.075 to 0.09)	<.001
-0.002 (-0.003 to -0.002)<.001Duration of AI use, weeks	0.004 (-0.115 to 0.123)	.948	-0.058 (-0.078 to -0.037)	<.001
Height, cm Duration of AI use in weeks x number of conversations	0.03 (-0.02 to 0.077)	.244	0.044 (0.035 to 0.053)	<.001
Baseline weight, kg	0.02 (-0.01 to 0.036)	.187	-0.008 (-0.012 to -0.004)	<.001
Number of conversations with the AI	-0.008 (-0.013 to -0.004)	<.001	-0.002 (-0.004 to 0.001)	.144
Number of meals logged	-0.012 (-0.020 to -0.004)	<.01	-0.035 (-0.039 to -0.031)	<.001
Healthy meals logged	-0.018 (-0.030 to -0.007)	<.01		
Unhealthy meals logged	-0.055 (-0.114 to 0.005)	.072	0.088 (0.068 to 0.107)	<.001
				

^a^Regression weighted by number of entries per user.

^b^Male-Female difference assessed using the Tukey-Kramer honestly significant difference test.

**Table 5:** User trust survey results.

Question	Mean	Standard deviation	Calculated scores
SS (n=70)	7.9	2.1	87^b^
NPS (n=76)	8.3	2.3	47^c^
DS (n=70)	6.7	3.2	68^d^
HOS^a^ (n=57)	NA	NA	60^e^

^a^The HOS was assessed and calculated from a rating scale (“Much worse,” “Somewhat worse,” “Exactly the same,” “Somewhat better,” and “Much better”), so mean and standard deviation could not be calculated.

^b^Percentage of users who rated satisfaction as 6-10 on a scale of 0-10.

^c^Percentage of detractors (score 0-6) subtracted from the percentage of promoters (score 9-10) [43].

^d^Percentage who rated disappointment if the HCAI were not offered as 6-10.

^e^Percentage of users who responded their health was “Much better than before” or “Somewhat better than before.”

The number of meals logged was significantly correlated with number of conversations. For every additional conversation, users logged approximately 0.6 additional meals (*R*^2^=0.9, *P*<.01). The most number of average meals (33% [22.4/68]) were logged when users were in Module 2.

### Artificial Intelligence Acceptability and User Satisfaction

The in-app user trust survey had a 100% response rate. The average scores for Questions 1 (satisfaction in program), 3 (disappointment if not offered), and 4 (health outcome) were 7.9, 8.3, and 6.73, respectively. The average SS, NPS, DS, and HOS scores were 87, 47, 68, and 60, respectively (Table 5).

## Discussion

### Principal Results

This study showed that users of a conversational AI can lose a magnitude of weight comparable to that achieved with lifestyle change programs with live components among individuals with high diabetes risk. This suggests a value in investigating the potential for patients to use AI to effectively drive positive changes in lifestyle behaviors associated with preventing the development of diabetes. In this study, use of the HCAI was associated with average weight loss of 2.4 kg or 2.4%, which is comparable to a loss of 2.32 kg reported in a meta-analysis of 22 lifestyle intervention studies among individuals with risk factors for diabetes [49]. In the SCALE trial, average weight loss was 2.8 kg (2.6% of body weight) among participants in the lifestyle plus placebo group, who received weekly individual or group dietary and exercise counseling [50].

A separate review examined the results of trials of Web-based interventions for weight loss among adults [51]. The average weight loss among participants who completed the respective interventions in the studies highlighted in this review ranges from 1.3%-9.2% of starting weight; the range is 1.3%-3.8% when excluding the three most effective weight loss interventions, which all included human components. These values are comparable to the amount of weight loss (2.3% of baseline) observed in our study, although unlike in our study, most of the studies include human components in their interventions.

The weight loss achieved in this study has further implications for public health when considering the Finnish National Diabetes Prevention Program, a community-based program with one-on-one counseling visits or group sessions covering topics such as weight loss, diet quality, and exercise. Despite the in-person component of the program, average weight loss among 919 participants was 1.2%, which is less than the weight loss recorded in our study without an in-person component or the costs associated with it [52].

Also of note is that most users registered for HCAI between August and October 2016, so a significant proportion of program participation and associated weight loss occurred over the holiday season. This is a time when 51% of annual weight gain is estimated to occur. About half of adults gain 1% of body weight [53], and 14% of overweight and obese individuals gain at least 2.3 kg more than normal weight [54,55].

The amount of weight loss in this study may be clinically significant for diabetes risk. Weight loss of 1 kg can lower diabetes risk by 16% [6], and another study found that losing 5 kg is associated with a 50% decrease in risk [7]. Losing as little as 5% of excess body weight improves insulin sensitivity [56]. Even preventing weight gain is important, since gains of 5-7.9 kg and at least 8 kg body weight raise relative risk by 1.9 and 2.7, respectively [7].

The HCAI users recorded improvements in dietary patterns, as percentage of healthy meals logged increased by 31% and unhealthy meals decreased by 54%. This shift in meal quality indicated increased consumption of healthy food compared to unhealthy foods. The result is another potential decrease in diabetes risk, since even small shifts in diet composition can have significant impacts on diabetes risk [57,58].

This study also showed that conversational AI delivered via mobile phone app can have high acceptability among users. The NPS was 47, compared to the health industry average of 18, with the industry leader, Kaiser Permanente, achieving a score of 43 [59]. User satisfaction was 87%.

### Comparison With Prior Work

Previous studies have investigated the effectiveness of Web-based programs and found mixed results. A recent systematic review of systematic reviews concluded that Web-based programs had consistently better results than no program but were sometimes less effective than traditional, in-person weight control programs [60]. The magnitude of weight loss reported in this study is comparable to that found in other studies reporting weight loss among mobile phone app users. For example, 77.9% of users reported weight loss while using a health and fitness mobile phone app with weight, food, and physical activity tracking features [61]. Another study investigated the effects of a mobile phone‒based health coach on weight loss and health behaviors among overweight or obese young adults [62]. Those who were assigned to the intervention group lost an average of 1.8 kg compared to a gain of 0.3 kg among those in the control group. Notably, in contrast to our study, this study included an in-person counseling session at baseline for both groups, with the intervention group receiving a second 40-minute session at baseline.

To be able to accurately claim to be an option for increasing lifestyle change program access to patients, an AI lifestyle coach must achieve health outcomes comparable to those of traditional in-person programs, while being less costly. The weight loss of 2.4% observed in this study is comparable to the 2.3% weight loss reported in a Centers for Disease Control and Prevention Web-based lifestyle modification DPP program among individuals at risk for diabetes [49]. When comparing our study’s results to those of the original DPP study, the 2.4% weight loss was greater than the 2.1% in the metformin group, which had a 31% lower incidence of diabetes during follow-up [63]. The intensive lifestyle modification group in that study had a 5.6 kg weight loss and 58% lower incidence of diabetes, but they received 16 one-on-one lessons in the first 24 weeks, with additional one-on-one and group sessions after that. In contrast, our intervention required no live assistance in setting up or using the health coach.

This is only an early study, but it is important to determine which components of the health coaching app may have contributed to weight loss among users. While the app included logging and tracking features, the program also included health coaching that included educational components and behavior change support based on CBT. A previous study [64] found no extra weight loss, compared to a control group, among users of a popular calorie-counting app with weight tracking and physical activity logging but without health coaching. This implies that the health coaching aspects of the HCAI app may have had a significant impact on weight loss.

The AI was found to have high acceptability among users, which can improve retention in weight loss programs [65]. Previous studies have documented the acceptability of telehealth interventions for weight management. For example, one study among overweight participants found that amount of weight loss and program satisfaction was as high in a telehealth program as in a traditional program, and furthermore, participants rated the telehealth program as more convenient [66].

### Limitations

A study limitation was its lack of control group for direct comparison. However, it can be assumed that without a weight loss intervention, a control group would not lose weight and might gain weight since the average annual weight gain among American adults is 0.5-1 kg [67]. Another limitation was that weight loss and dietary intake were self-reported, although evidence suggests these data can be considered sufficiently valid [42,43,46]. Counterbalancing these limitations of the current dietary assessment method is user ability to avoid the potential for memory bias, as could be a concern when conducting weekly or other periodic in-person diet assessments. Reporting dietary intake via self-entry into the HCAI rather than to a live person also avoids biases stemming from social desirability, which are a common source of inaccuracies in dietary assessment [68]. Since the HCAI both guided participants on food and beverage choices and assessed participants’ meal quality, it is also possible that participants modified their food choices based on app feedback or deliberately misreported dietary intake to record “healthy” meals. Inaccuracies in self-reported weight can be avoided in the future as the HCAI incorporates the use of cellular weight scales so user weights are automatically recorded.

The scarcity of demographic information collected from users could be seen as a limitation of the study, since it is unknown which subpopulations would be likely to achieve similar results if they were to use the app in the future. However, the fact that the average participant lost weight despite lack of screening based on demographics suggests a wider applicability of the app in weight loss interventions.

Because this study was observational and not experimental, another limitation was its inability to determine causality. Participants who had at least one conversation with the HCAI in at least 4 different weeks lost 2.4% of baseline body weight on average, but it was not determined whether app use caused weight loss, or whether weight loss was caused by lifestyle changes resulting from app use or from other causes, or whether weight loss resulted from another cause that was not investigated in this study. Furthermore, because participants were not screened based on weight loss intentions, nor asked follow-up questions regarding behaviors related to weight loss, it is possible that some weight loss could have resulted from causes unrelated to HCAI use or lifestyle changes encouraged by the HCAI. It is conceivable, for example, that participants took weight loss medications or underwent bariatric surgery during the study period. Future research should include an experimental study that includes data collection surrounding possible confounding or other factors related to weight loss.

The AI automatically tracked physical activity according to any motion detected by mobile phone sensors, and users could log their activity manually. Inaccuracies could result if users completed physical activity bouts without carrying their phones (ie, activity was not automatically detected and recorded) and users neglected to manually input these bouts, or if users double-logged physical activity; that is, if their workout was detected and recorded automatically and they separately entered it manually. Similarly, sleep duration was detected and recorded automatically, but users could add, modify, or delete data.

Another limitation was the potential for incomplete or incorrect classification of foods and therefore meals. This could be due to missing foods in the Lark food database or to incorrect classification of foods as healthy, unhealthy, or neutral when users entered ambiguous foods (eg, “chicken salad” could comprise mayonnaise and chicken and be “unhealthy” or comprise chicken and lettuce and be “healthy”).

### Applicability in Chronic Disease Management

The cost of chronic diseases comprises 75% of health care costs in the United States [69], but risk reduction is possible through lifestyle changes. Primary care support is limited, and supplementation is needed. One study found a median primary care visit length of 15.7 minutes, which included covering a median of 6 topics. Typical length spent on each topic was 5 minutes for the longest topic, and 1.1 minutes for the others [70]. These figures suggest that mHealth technology, such as conversational AI in mobile phone apps, could fulfill a need by complementing in-person health care providers in supporting behavior change. This is supported by the results of a systematic review that concluded that telemedicine interventions for chronic disease management can lead to improved health outcomes and lower costs of care and have high user acceptability [71]. Table 6 compares selected characteristics of in-person coaching and HCAI.

**Table 6:** Comparison of selected characteristics of in-person coaching and health coach artificial intelligence.

Characteristic	In-Person Coaching	HCAI
Number and frequency of coaching sessions	Sessions can be limited to a certain number per day, week, or program.	Sessions are unlimited.
Need to schedule appointments	Appointments for coaching sessions may be required.	Users can initiate coaching sessions without an appointment.
Coaching availability	Coaching may be available only during set hours	Coaching is available anytime: day, night, or weekends.
Cost of coaching	Insurers, healthcare providers, and/or patients must pay salaries and/or per-session costs of health coaches.	There is no salary or additional per-session cost associated with HCAI.
Patient level of comfort	Live coaches can be intimidating.	Patients can identify personal challenges without fear of shame or judgement by the HCAI

The HCAI could potentially improve access to weight loss behavior change interventions. Telemedicine interventions, including those using mobile phones as a means of delivery, can be effective in reaching underserved populations, such as isolated rural communities and inner-city communities without sufficient providers compared to the number of patients [72]. Telemedicine interventions also have the built-in advantage of potentially being lower in cost than in-person or even remote appointments with live providers. The cost of labor associated with health care is estimated to be US $24 per visit to a primary care provider [73], while there is no additional cost per interaction with the HCAI. Patients installed and used the HCAI on their own after receiving the link, and the health coaching sessions did not require any time commitment from providers.

### Conclusions

#### Compassionate Care for Weight Loss and Behavior Change

The HCAI was designed to promote weight loss and healthy lifestyle behaviors in a compassionate experience using conversational AI. It included elements of CBT interventions with in-the-moment responses based on user input including user-initiated conversations about feelings and accomplishments, and user-entered behaviors including weight, food consumption, and physical activity. The HCAI takes a holistic approach, providing both strategic suggestions and emotional support, and aiming to make users feel valued. For example, it responds to a challenge, such as guilt over overeating, by providing an idea about how to approach the situation in the future (“Just let this feeling give you insight/Into how you might want to do things differently next time”) and reminding users of their worthiness (“You are a wonderful and worthy human being, deserving of the best treatment you can give yourself”). The HCAI design also considers the challenge of long-term maintenance of weight loss, since an estimated 80% of those who lose at least 10% of weight loss for at least a year eventually experience regain [69]. To promote long-term weight loss, this health coach supports self-efficacy, healthy behaviors such as regular weigh-ins and increased physical activity, and a multidisciplinary approach, which is linked to better success [74].

#### Future Work

As seen in this study, technology for fully automated health coaching AI is available for real-life applications. Results from this study showed that participants lost weight while using the HCAI, which implies a potential for the HCAI to aid patients and providers in losing excess weight and improving health behaviors. The study also demonstrated the ease of use of the app, since participants received no assistance in installing or using the app, and its engagement and acceptability among overweight and obese participants (Table 5).

Additional work is underway or being planned to further investigate health coaching AI and its roles in chronic disease management. Health coach AI apps similar to the weight loss‒focused HCAI in this study have been developed and are being used for prediabetes management and for diabetes prevention and management. A version for managing pre-hypertension is also under development.

Current work includes a randomized controlled trial to investigate effects of the AI on aspects of chronic disease management including weight control, diet quality, medication adherence, and home blood pressure monitoring among individuals with pre-hypertension. Another planned study is a retrospective study among individuals with prediabetes who use a version of the health coach that is a DPP. Outcomes include weight loss and self-efficacy.

This study demonstrates AI’s potential to provide compassionate care that is associated with weight loss, increased healthy lifestyle behaviors, and user trust that can reduce diabetes risk.

## Abbreviations

AI: artificial intelligence
BMI: body mass index
CBT: cognitive behavioral therapy
DPP: Diabetes Prevention Program
DS: disappointment score
HCAI: Lark Health Coach Artificial Intelligence
HOS: health outcome score
NPS: net promoter score
SS: patient satisfaction score
T2D: type 2 diabetes mellitus
